# X-ray computed microtomography of *Megachirella wachtleri*

**DOI:** 10.1038/sdata.2018.244

**Published:** 2018-11-06

**Authors:** Tiago R. Simões, Michael W. Caldwell, Mateusz Tałanda, Massimo Bernardi, Alessandro Palci, Oksana Vernygora, Federico Bernardini, Lucia Mancini, Randall L. Nydam

**Affiliations:** 1 Department of Biological Sciences, University of Alberta, Edmonton, Alberta T6G 2E9, Canada; 2 Department of Earth and Atmospheric Sciences, University of Alberta, Edmonton, Alberta T6G 2E9, Canada; 3 Department of Palaeobiology and Evolution, Faculty of Biology, University of Warsaw, Żwirki we Wigury 101, 02-089 Warsaw, Poland; 4 MUSE - Museo delle Scienze di Trento, Corso del Lavoro e della Scienza 3, Trento, 38123 Italy; 5 School of Earth Sciences, University of Bristol, Bristol, BS81RJ, UK; 6 College of Science and Engineering, Flinders University, Adelaide, South Australia 5042, Australia; 7 South Australian Museum, North Terrace, Adelaide, South Australia 5000, Australia; 8 Museo Storico della Fisica e Centro di Studi e Ricerche “Enrico Fermi”, Piazza del Viminale 1, 00184 Roma, Italy; 9 The “Abdus Salam” International Centre for Theoretical Physics, Strada Costiera 11, Trieste, 34151 Italy; 10 Elettra - Sincrotrone Trieste S.C.p.A., SS 14, Km 163.5, Area Science Park, Basovizza, 34149 Trieste, Italy; 11 Department of Anatomy, Arizona College of Osteopathic Medicine, Midwestern University, 19555N. 59th Dr., Glendale, AZ 85383, USA

**Keywords:** Palaeontology, Herpetology, X-ray tomography, Phylogenetics

## Abstract

Understanding the origin and early evolution of squamates has been a considerable challenge given the extremely scarce fossil record of early squamates and their poor degree of preservation. In order to overcome those limitations, we conducted high-resolution X-ray computed tomography (CT) studies on the fossil reptile *Megachirella wachtleri* (Middle Triassic, northern Italy), which revealed an important set of features indicating this is the oldest known fossil squamate in the world, predating the previous oldest record by ca. 75 million years. We also compiled a new phylogenetic data set comprising a large sample of diapsid reptiles (including morphological and molecular data) to investigate the phylogenetic relationships of early squamates and other reptile groups along with the divergence time of those lineages. The re-description of *Megachirella* and a new phylogenetic hypothesis of diapsid relationships are presented in a separate study. Here we present the data descriptors for the tomographic scans of *Megachirella,* which holds fundamental information to our understanding on the early evolution of one of the largest vertebrate groups on Earth today.

## Background & Summary

X-ray CT scanning technology at the micron scale (μCT) is revolutionizing the acquisition and analysis of anatomical data for both extant and fossil species (for examples, see^1–4^). In the case of paleontological data, CT scanning enables faster, safer, and more complete data acquisition compared to the usually slow process of rock matrix removal in traditional fossil preparation. Furthermore, small-bodied specimens may be too delicate for extensive mechanical or chemical preparation, and as a result, only part of the specimen can be directly observed. X-ray CT scanning thus becomes fundamental for extracting anatomical data from the rare available fossils of early lepidosaurs^5–11^.

In order to improve the current understanding of the early evolutionary history of squamates and other diapsid reptiles, we performed X-ray μCT scans of the fossil reptile *Megachirella wachtleri* (Middle Triassic, Italian Alps^12,13^), which is one of the few well-preserved and articulated lepidosaur species from the Triassic. Additionally, we assembled the largest species level phylogenetic data set of diapsid reptiles, the first one with extensive sampling of squamates (represented by both morphological and molecular data). Our major goals were to obtain the largest and most accurately sampled data library to understand the divergence of lepidosaurian reptiles from other reptile lineages, the earliest stages of the acquisition of squamatan features, and the time of origin of the major diapsid reptile clades (presented in a separate study^14^).

The μCT data of *Megachirella* provides fundamental new morphological information to elucidate the patterns and processes of early squamates evolution, and early diapsid reptile evolution, broadly speaking. Therefore, the data assembled by us will be useful to herpetologists, paleontologists and evolutionary biologists with research programs investigating broad-scale and deep time problems in the evolution of reptiles.

## Methods

### Microfocus X-ray computed tomography

*Megachirella wachtleri* is preserved on the surface of a dense, thick and large rock slab (approximately 160 mm long × 120 mm at its widest point × 30 mm deep, weighting 721 grams). The shape and density of the slab make the μCT analysis of the fossil not trivial. Despite the residual noise remaining in the reconstructed images after the procedures adopted for denoising and artefact reduction, a relatively good contrast was obtained between osteological remains and the surrounding matrix. Therefore, most of (although not all) osteological elements could be easily distinguished and separated from the rock matrix and from each other during segmentation.

The holotype of *Megachirella* was analysed by microfocus X-ray computed tomography (μCT) at the Multidisciplinary Laboratory (MLAB) of the “Abdus Salam” International Centre of Theoretical Physics (ICTP) in Trieste (Italy), using a system specifically designed in collaboration with Elettra Sincrotrone Trieste (Trieste, Italy) for the study of paleontological and archaeological materials^15^.

X-rays are produced by a Hamamatsu microfocus source L8121-03 (150 kV maximum voltage, 500 μA maximum current, 5 μm minimum focal spot size). Two linear translation axes and a high-resolution rotation stage by Aerotech allow precise movements of the samples. The detector used for this study is a Hamamatsu CMOS flat panel model C7942-SK25, featuring a 50 microns pixel size and an active area of 112 × 118.4 mm^2^. This system design allows different detectors and sample stages to be easily installed depending on the specific scientific application.

The devices mentioned above are mounted on a versatile mechanical setup, which can be disassembled and transported. In fact, the design philosophy was to develop an instrument that could be transported and installed in museums or in general where important finds are located to perform *in-situ* analyses of precious samples. Furthermore, this setup has been designed to allow a flexible adjustment of source-to sample and sample-to-detector distances in order to be able to exploit phase-contrast effects if an appropriate detector is used. The integration of software to control the X-ray tube, detector and sample stage motors for the μCT scans was custom-developed.

This system allows the investigation of relatively large objects (with dimensions up to a maximum width of ca. 20 cm and a weight up to 15 kg) at 40–50 μm voxel size. Smaller objects can be also studied achieving an isotropic voxel size of up to 5 μm. Using this system, the ICTP-Elettra laboratory successfully investigated several paleontological, paleoanthropological and archaeological materials—e.g. Bernardini *et al.*^16^; Neenan *et al.*^17^; Holgado *et al.*^18^; Aráez *et al.*^19^; Di Vincenzo *et al.*^20^; Fernández-Coll *et al.*^21^.

### X-ray μCT acquisition parameters

An X-ray μCT acquisition of the complete specimen was carried out by using a sealed X-ray tube at a voltage of 150 kV, a current of 100 μA and setting a focal spot size of 20 μm. The X-ray beam was filtered by a 1.5 mm-thick aluminium absorber. A set of 2400 projections of the sample was recorded over a total scan angle of 360° by a flat panel (see previous section) detector with an exposure time per projection of 2 s (Table 1). The tomographic axial slices were reconstructed using the commercial software Digi XCT (DIGISENS) (https://www.digisens3d.com/digi-xct/) in 32-bit tiff format and an isotropic voxel size of 42.5 μm). Additionally, the cranium of *Megachirella* was re-analysed (voltage 150 kV, current 100 μA, 1 mm-thick copper filter, 3 s exposure time per projection, and acquiring 2400 projections over 360°) setting an effective pixel size of 18 μm and reconstructed using the same software in order to achieve a higher spatial resolution (Table 1).

### Segmentation procedure

The reconstructed slices for the whole body and skull scans in 32-bit tiff format were converted into 8-bit tiff format using the software ImageJ in order to make total file size feasible to be processed by 3-D rendering software, and so that segmentations could proceed without reaching the limits of the available RAM memory and video graphics.

**Figure 1:**
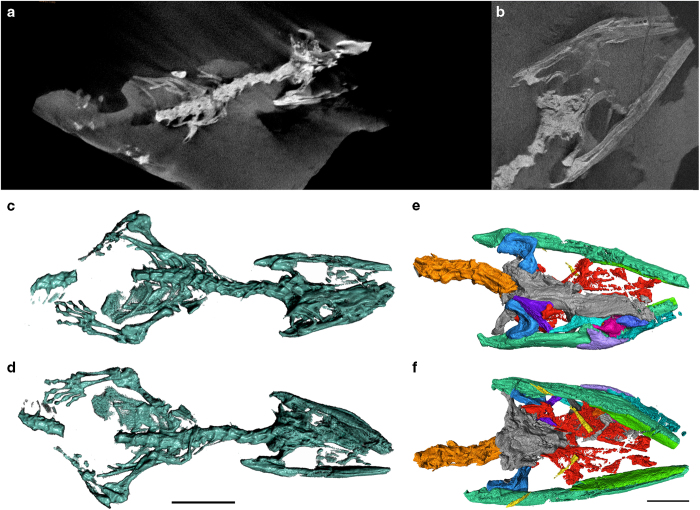
Segmentation procedure of the CT scan data of *Megachirella wachtleri*. (**a**) Sample slice of the whole-body scan of *Megachirella*. (**b**) Sample slice of the head-only scan of *Megachirella*. Each scan is composed of hundreds or thousands of slices that were subsequently used for individual segmentation procedures. (**c**) Whole skeleton after segmentation in dorsal view. (**d**) Whole skeleton after segmentation in ventral view. (**e**) Skull and mandibles after segmentation of individual components of the head in dorsal view. (**f**) Skull and mandibles after segmentation of individual components of the head in ventral view.

The skull of *Megachirella* was segmented in Avizo v. 9.0 (Thermo Scientific™). The stack of Tiff images (1913 slices, 1728 × 651 pixels) was imported into the software and the Threshold Tool was used to remove as much rock matrix as possible. However, because the density of some of the most delicate bones (e.g. hyoid bones) was very similar to that of the rock matrix, the threshold was chosen so that the former elements would not be affected (Fig. 1). This left some rock matrix that had to be removed via manual segmentation slice by slice.

Following this preliminary step, the individual skull bones, where possible, were segmented using the Lasso and Brush tools in Avizo v. 9.0 and assigned to different materials. The geometric boundaries of the bones were identified by looking at sections of the skull in three orthogonal planes. Bones that could not be separated due to excessive crushing and unclear boundaries were assigned to the same general material depending on the skull region (e.g. most palatal bones, most bones from the braincase). The segmented elements were then rendered as a surface (through Generate Surface and Surface View modules in Avizo v. 9.0).

Segmentation of the whole body was executed using Dragonfly v. 1.0 (Object Research Systems (ORS) Inc.™). The stack of Tiff images (1097 slices, 2025 × 921 pixels) was imported into the software and most of the rock matrix was initially removed using quick segmentation (via the “define range” thresholding tool). Subsequently, the advanced analysis tool was utilized in order to remove most of small matrix objects remaining in the quickly segmented object. The third stage consisted of manually removing what was left of the rock matrix slice by slice, with the aid of the 3-D segmentation tool with “define range” activated. The final 3-D visualization of the whole skeleton was also rendered in Dragonfly v. 1.0 (Fig. 1).

## Data records

Data record 1— The X-ray μCT data reported in this manuscript has been deposited in a Figshare repository (Data Citation 1). The data comprises the tomographic images (slices) of two separate scans: one from the whole body of *Megachirella* and one focused on the head of *Megachirella*. The files are in TIF format, and the metadata associated with the scanning procedures and experimental parameters, are available in the log file provided with the image files.

## Technical validation

The X-ray CT scanner is subject to regular maintenance. This reduces the chances of technical artefacts during the emission of X-rays and capturing of the images. Typical artefacts resulting from CT scanning can include streaking, shading and rings. To reduce some of these effects, ring artefact reduction and beam hardening correction have been applied in order to improve the quality of the reconstructed slices. As a result, no significant artefacts were detected in the resulting images.

Additionally, depending on the composition of the rock matrix where the osteological remains of fossil vertebrates are preserved, obtaining a significant contrast between fossilized bones and rock matrix can be extremely challenging. Therefore, besides the regular procedure of calibrating voltage, current, and exposure parameters during image acquisition in the CT scanner, contrast in the image slices was further enhanced using the freeware ImageJ^22^.

Information obtained from the μCT scans of *Megachirella* along with our personal observations of the holotype of this taxon were used for the re-description of this species and for the scoring of its anatomical data in the new phylogenetic data set provided by us (see supplementary information in Simões *et al.*^14^). It is important to note that *Megachirella* along with over 75% of other taxa in our phylogenetic data set were scored in the data matrix while observing the specimens in their respective collections. In our experience, this practise increases efficiency and accuracy during data scoring by depending less on the information provided by anatomical drawings, pictures, personal notes and the availability of μCT scan data.

## Usage Notes

The X-ray μCT scans data slices in TIF format can be imported into 2-D and 3-D image processing, analysis and visualization software packages, such as Dragonfly (http://www.theobjects.com/dragonfly/), Avizo/Amira (https://www.fei.com/software/amira-avizo/), VGStudio Max (https://www.volumegraphics.com/en/products/vgstudio.html), and *Pore3D* (https://github.com/ElettraSciComp/Pore3D)^23^ among others. This data set can be used to produce new segmentations or volume mesh files for additional analyses, such as finite element analysis.

## Additional information

**How to cite this article**: Simões, T. R. *et al.* X-ray computed microtomography of *Megachirella wachtleri*. *Sci. Data*. 5:180244 doi: 10.1038/sdata.2018.244 (2018).

**Publisher’s note**: Springer Nature remains neutral with regard to jurisdictional claims in published maps and institutional affiliations.

## Supplementary Material



## Figures and Tables

**Table 1:** X-ray μCT acquisition parameters used to scan *Megachirella wachtleri*.

**Anatomical part**	**DSO (mm)**	**DSD (mm)**	**Exposure time (seconds)**	**Voltage (kV)**	**Current (μA)**	**Filter**	**Projections**	**Voxel size (micron)**
Whole specimen	625	735	2	150	100	1.5 mm Al	2400	42.5
Head	225	625	3	150	100	1 mm Cu	2400	18.0
DSO: distance source – object; DSD: distance source – detector.								
